# Food Hygiene Practices of Rural Women and Microbial Risk for Children: Formative Research in Nepal

**DOI:** 10.4269/ajtmh.20-0574

**Published:** 2021-09-20

**Authors:** Om Prasad Gautam, Valerie Curtis

**Affiliations:** ^1^ London School of Hygiene and Tropical Medicine, London, United Kingdom;; ^2^ WaterAid UK, London, United Kingdom

## Abstract

Formative research was conducted in a rural hill setting in Nepal during April–June 2012 to inform the design of an intervention to promote safe food hygiene practices. A variety of methods underpinned by Behavior Centered Design theory and Hazard Analysis Critical Control Points was used to pinpoint key risk behaviors and their environmental and psychological determinants in 68 households with a mother having a child aged 6–59 months. These included video recordings, observation of daily routine, teach-the-researcher sessions, in-depth interviews, observations of actual behaviors, focus group discussions, motive mapping, microbiological assessment, and identification of critical control points. Physical settings, especially the kitchen, form a challenging environment for mothers, including family members in rural hill settings of Nepal to practice adequate food hygiene behaviors. Prevalent food hygiene practices of mothers were inadequate, leading to frequent exposure of young children to highly contaminated food, water, and milk. We identified six critical control points; of these, five needed improving. Determinants of these behaviors included physical and social environment as well as psychological brief and individual motives. Five key food hygiene risk behaviors are suggested for prioritization. While designing a food hygiene intervention package, consideration should be given to the physical, biological, and social environment, and immediate motives behind each practice should be taken into consideration while framing key messages. Creative and engaging activities should be designed around the motives of nurture, disgust, affiliation, and social status/respect.

## INTRODUCTION

Poor food hygiene is likely to be an important contributor to high rates of infectious diseases in resource-poor settings. According to some estimates, up to 70% of diarrheal episodes in developing countries are caused by pathogens transmitted through food.^1^^,^^2^ Children are particularly vulnerable to diarrheal infections during the weaning period^3^^–^^5^ when protection from breast milk reduces and complementary food is introduced. High rates of diarrheal infections contribute to problems of undernutrition,^6^^–^^8^ where a quarter of stunting can be attributed to a child having five or more episodes of diarrhea before 2 years of age.^5^ Environmental enteropathy arising from subclinical exposures to fecal pathogens^7^ may be a further cause of poor growth in children residing in unhygienic environments. Although poor food hygiene has often been implicated as a source of diarrhea pathogens in industrialized countries,^9^^–^^12^ there have been few studies of microbial food contamination, and even fewer studies on interventions to improve this situation, in the low-income countries that have the highest disease burdens.

Preparing and serving uncontaminated food is difficult in such contexts mostly because water may not be available on tap, sanitation facilities may be substandard or absent, surfaces are hard to clean, cleaning products are scarce, and refrigerators uncommon despite high ambient temperatures. Such studies as there are point to high levels of microbial contamination in foods fed to young children^3^^,^^13^^–^^18^ and food preparation as a risk factor for diarrheal diseases,^10^^,^^17^^,^^19^ including cholera.

Improving food hygiene is therefore a priority for infectious disease control. However, before we can intervene on the scale that is required, we need to better understand the behaviors that cause risk and their determinants. This formative research study thus used a variety of theory-based methods to investigate food hygiene behavior in a rural hill area of Nepal. The objective was to identify key food hygiene behaviors and their determinants to inform the design of an intervention for trial and potential scale-up.

## MATERIALS AND METHODS

### Study site and sampling.

The formative research was conducted in Kavre District, Nepal. Here, diarrhea is the second leading cause of child death, and around 41% children are stunted.^20^ The residents live on steep hills and subsist through small-scale agriculture and dairy farming. Their ethnic origins are mixed, with a majority (63%) belonging to the Hill Aadiwasi/Janajaati (Tamang) group with a Brahmin/Chhetri (28%) minority. Around 70% of households had piped water, and 44% practiced open defecation. Two wards that were not scheduled to be part of the intervention trial were randomly selected for formative research, and all 68 households with children aged 6–59 months in the two wards consented to participate in the study. Mothers in households with a child aged 6–59 months were invited to participate in the study as key respondents. Ethical approval was granted by the Research Ethics Committee of the London School of Hygiene and Tropical Medicine (LSHTM) (no. 6164) and the Nepal Health Research Council (NHRC).

### Conceptual frameworks.

The study used two conceptual frameworks to conduct formative research. To identify behaviors likely to cause risk of pathogen transfer, we used a modified version of the Hazard Analysis and Critical Control Points (HACCP) approach.^21^^–^^23^ This is a systematic approach to the identification, assessment, and control of food-related hazards.^21^ We used steps for the identification of hazards associated with food preparation, handling, and feeding; assessing risk; and determining points where critical control measures would be applied.

To understand food hygiene–related behavior in context, we used Behavior Centered Design, a systematic approach to designing behavior change interventions developed at LSHTM.^24^^–^^27^ The approach has five steps: A-Assess, B-Build, C-Create, D-Deliver and E-Evaluate. Formative research was the B-Build step in the process; the approach pinpoints key behaviors; seeks to identify causes for the behaviors that are psychological (habitual, motivated, or planned), bodily, and environmental (social, biological, and physical); and pays attention to behavioral settings, which are akin to theaters of performance of regularly occurring routine behaviors with roles, scripts, props, norms, and purposes.^28^

### Study methods and instruments/tools.

The study used microbiological methods to investigate food contamination and anthropological and consumer research techniques to understand risk behaviors in social and physical contexts. Four field data collectors were locally recruited and trained to collect data using various tools. In addition, five local women from different castes/ethic groups with similar academic backgrounds were recruited and trained to use hand-held cameras to ensure cultural sensitivity, and they recorded mothers preparing, cooking, handling, storing, feeding, and reheating child foods. To reduce observer bias, reactivity, and potential alternation in behavior, participants were told that we are monitoring their daily routine and child’s interference on their daily routine using various tools, including video observations. Food, water, milk, and Jad samples were collected using four trained laboratory technicians (sample collectors), and samples were analyzed at a local laboratory in Kathmandu using four trained laboratory technicians and a microbiologist. Following are summaries of the methods and instruments/tools used in the study.

### Basic characteristics.

In-depth interviews and household surveys were carried out with all 68 mothers concerning their socio-demographic characteristics, access to basic services, common child food, the current source of knowledge, common infections, etc.

### Microbiological methods and instruments/tools.

#### Microbial assessment.

Total coliform (TC) count and *Escherichia coli*, the WHO-recommended indicator organisms for measuring fecal contamination,^29^ were quantified in commonly used child food, water, milk, and Jad (a local alcoholic brew) from 30 households randomly selected from those who agreed to participate. One hundred five food samples were collected at four different stages (30 immediately after cooking, 30 during feeding, 30 after 5 hours of storage, and 15 immediately after reheating). Thirty samples of ready-to-serve water, 13 of milk, and 12 of Jad were collected, transported, homogenized, inoculated, and incubated, and results were interpreted using standard operating procedures and appropriate media (PetriFilm; 3M, St. Paul, MN).

#### Hazard analysis and critical control points.

A food flow diagram (food supply chain from collection of raw materials to feeding) was developed based on observed patterns of food preparation, storage, and feeding in 68 households. Potential sources of hazards and possible re/contamination were documented through structured observation and using microbe data. Critical control points were identified based on their role in bacterial destruction, survival, and or propagation. Behaviors were prioritized for intervention based on whether control points acted as barriers and whether respective behavioral actions could be applied as control measures.

### Behavioral methods and instruments/tools.

#### Observation.

The physical environment and levels of cleanliness were assessed in 68 households by observation using standardized definitions and checklists.

#### Video recordings.

Food preparation behavior was filmed in 30 randomly selected households within their originally recruited 68 households. Local women were trained to use hand-held video cameras to follow all aspects of child food preparation, cooking, handling, storing, feeding, and reheating. Filming was continuous for 2–3 hours, except when privacy was required.

#### Observation of daily routines.

The daily work routine of 30 mothers starting from early morning (wake-up) to the end of the day (going to bed) was recorded using the Day in Life Analysis (DILO) tool. This tool helps to assess whether food hygiene behaviors are part of their daily routine work. Specific actions during cooking time were assessed using the Moment in the Life Analysis (MILO) tool.

#### “Teach the researcher” sessions.

Five mothers were asked to teach the researcher how to cook their child’s food while the researcher observed closely the mother’s actions during food preparation, cooking, feeding the child, storage, and reheating.

#### Focus group discussions.

Nine focus group discussions (FGDs) with mothers and two with grandmothers were conducted to identify commonly used child foods to understand the food chain supply mechanism and key barriers and to identify socio-cultural practices around food preparation, handling, storage, feeding, and reheating.

#### Motive mapping.

Eleven motivation mapping sessions were performed with 68 mothers to assess their immediate motives for carrying out key practices. In each motivation mapping session, seven different pictures demonstrating common motives (attraction, nurture, disgust, status, respect, affiliation, purity, and disease) were shown, and stories were articulated around those pictures. After discussion, mothers were asked to rank the pictures according to how likely these were to motivate them to practice key behaviors.

### Data analysis.

#### Microbiological analysis.

Fifty grams of food in each stage, 250 mL water, 50 mL milk and 50 mL of Jad were collected and transported to the laboratory maintaining a temperature below 6°C. Total coliform (TC) and *E. coli* colony counts per gram of food (cfu/g of food), cfu/100 mL of water, cfu/mL of milk and Jad were recorded in the laboratory. 3M-PetriFilm, *E. coli*, and coliform count plates^30^^,^^31^ were used to detect TC and *E. coli* in food and milk samples. Membrane filters (0.45 μm pore size) and eosine methylene blue agar media were used for water and Jad samples. Colony counts were log-transformed (log_10_) to compare the mean counts at four different stages for food. The counts of TC and *E. coli* were categorized as < 10 cfu/g, 10–100 cfu/g, and > 100 cfu/g of food. The temperature of food samples and pH of milk, water, and Jad samples was recorded.

#### Behavioral analysis.

Interviews and FGDs were recorded and transcribed, and video recordings were watched to identify key behavioral actions and missed opportunities to control food contamination. The data from behavior observation, the teach the researchers session, and the motives session were categorized under each behavior of interest initially. Food follow diagrams were used to visualize likely hazards and identify critical and behaviural control points. Quantitative data (observations, survey, motive mapping) were entered and analyzed using SPSS statistics 19 (IBM, Armonk, NY). The findings were organized following the categories of behavioral determinants in the BCD checklist.^24^ Behavior (planned, motivated, and habitual) and environmental (social, physical, and biological) settings and other relevant findings are presented under specific themes, and summary statistics are presented as frequencies.

## RESULTS

### Household and participant characteristics.

**Figure 1.**
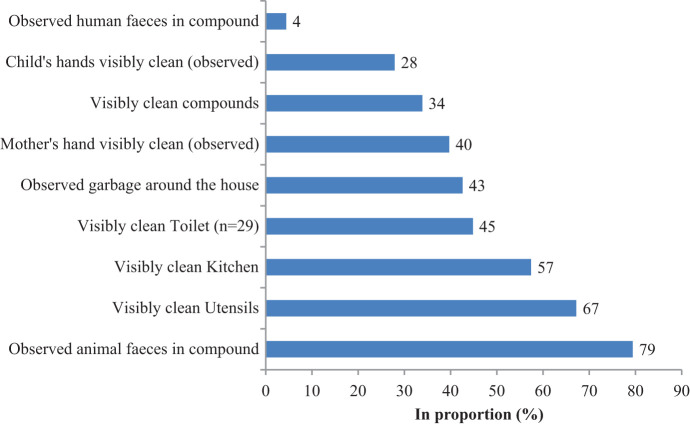
Environmental cleanliness during observations (*N* = 68). This figure appears in color at www.ajtmh.org.

The social and demographic characteristics of the study population are presented in Table 1. Respondents ranged in age from 17 to 43 years (mean: 28 years). The majority of the study participants were Tamang (75%), followed by Brahmin/Chhetri (19%) and Dalit (6%). The majority of the mothers had no or informal education, and the majority of households earned less than $100 per month. Most of the respondents were either housewives (50%) or dependent on agricultural work (27%). There were 84 children aged 6–59 months in the 68 households. Ninety-four percent of households had soap available (mostly laundry soap). Figure 1 highlights the environmental settings and cleanliness.

**Table 1:** Social and demographic characteristics of the participants and households (*N* = 68)

Variable	Mean (range)	Variable	Frequency, *N* (%)
Mother’s age	28 (17–43) years	Main source of household income	
Family members per house	7 (3–18) members	Agriculture	39 (57)
Children’s age (*N* = 84)	28 (6–58) months	Self-employment	6 (9)
		Manual labor	9 (13)
Education level of mothers, *N* (%)		Business/shop	3 (4)
None	18 (27)	Others	11 (16)
Informal	32 (47)	Monthly household income (NRs)	
Primary	7 (10)	< 1,000	6 (9)
Secondary	9 (13)	1,000–5,000	33 (49)
Higher secondary	2 (3)	5001–10,000	18 (27)
Cast/ethnicity of mothers		> 10,000	11 (16)
Brahmin/Chhetri/Thakuri	13 (19)	Main source of drinking water	
Hill Aadiwasi/Janajaati	51 (75)	Piped water in residence	27 (40)
Hill Dalit	4 (6)	Piped water to tap in yard, plot	33 (49)
Religion		Surface water	8 (12)
Hinduism	18 (27)	Households with toilet (observed)	
Buddhism	50 (74)	No	39 (57)
Occupational status of mothers		Yes	29 (43)
Housewife	34 (50)	Households with soap (observed)	
Unskilled labor	8 (12)	No	4 (6)
Agriculture	18 (27)	Yes	64 (94)
Teacher	1 (2)	Main source of cooking fuel	
Business	3 (4)	Firewood	64 (94)
Others	4 (6)	Kerosene	3 (4)
Households with refrigerator (observed)		Gas	1 (2)
No	67 (99)		
Yes	1 (1)		

NR = Nepalese rupee.

### Behavior related to food hygiene, its determinants, and level of microbes in food.

#### Daily routine of mothers,

Most mothers reported following a similar daily routine of rising at 5 AM, followed by defecation, fetching water, lighting the fire, cleaning the animal shed, feeding the animals, sweeping inside and outside the house, preparing tea, and feeding snacks (light breakfast) to children. This was followed by either domestic or field work (∼3 hours). Though cooking time varied by caste group, the majority of the mothers prepared lunch (the first main meal) between 9 and 11 AM. Most mothers (82%) needed at least 1 hour to prepare food. After feeding the child and eating, afternoon work included domestic tasks such as sweeping, laundry, feeding and caring for animals, or work in the field (∼3–4 hours). Snacks given to children in the afternoon mostly comprised leftovers of food cooked in the morning, often stored in the same cooking vessel or in a bowl or plate near the cooking area. Half of the mothers mentioned having some leisure time in the afternoons. Most mothers cooked dinner between 6 and 7 PM and then fed their children the freshly prepared food. After a short leisure or tidying-up period, the day ended at around 8–9 PM for all mothers. Most mothers did not report food hygiene behaviors as a part of their daily routine, such as washing hands with soap before feeding, reheating food, cleaning service utensils, and treating water.

#### Child food.

According to mothers during in-depth interviews and FGDs, female children are weaned at 5 months and male children at 6 months in rural Nepal. Weaning starts with a rice feeding ceremony (pasne), mostly using a sweetened rice pudding. Following this event, the majority of the mothers/caregivers reported feeding their children the same food that adults and older children consume daily. The majority of the households served solid food to children (only 20% said that they gave semi-liquid food to children under 24 months). Table 2 shows the types of commonly fed food to young children in Nepal. All mothers said that they also offered water to their children to drink and sometimes milk (43%); several Tamang families also offered Jad. Liquid foods included milk and water, semi-solid foods included Jaulo (a rice-based porridge), and the main solid food was rice with pulses and/or vegetable curry. Other foods included dhindo (maize- or wheat-flour porridge), roti (maize- or wheat-flour flatbread) with dal (pulses), lito (roasted rice-maize or millet-flour with ghee and sugar), khichari (rice, pulses, turmeric powder, and vegetables), fruits (banana, orange, etc.), boiled eggs, and breast milk.

**Table 2:** Types of food fed to young children (*N* = 84 children)

Age group	Food type (commonly used food), *N* (%)			Total
	Liquid	Semi-solid	Solid*	
6–23 months	1 (2)	8 (19)	33 (79)	42 (100)
≥ 24–59 months	0 (0)	0 (0)	42 (100)	42 (100)
Total	1 (1)	8 (10)	75 (89)	84 (100)

* Children eating solid food also eat liquid and semi-solid food.

#### Food preparation and cooking.

**Figure 2.**
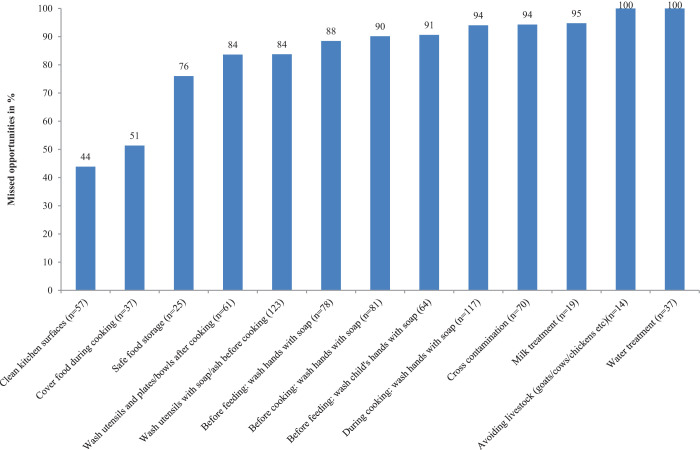
Observed missed opportunities to practice correct food hygiene behaviors (data from video analysis). This figure appears in color at www.ajtmh.org.

Our data obtained through observation, video observation, and daily routine analysis confirm that mothers or grandmothers generally cooked for and fed children. Though foods were usually prepared twice a day, children (6–23 months) were fed on average four to five times a day; all households therefore fed stored or leftover food to children. Mothers were multitasking while cooking, making cross-contamination of food likely; such tasks included feeding animals, sweeping or wiping the kitchen, washing utensils, using the toilet, doing laundry, cleaning the child’s bottom, breastfeeding, and wood or water collection. Other behaviors likely to cause contamination were tasting curry using fingers instead of a spoon, grinding spices without cleaning the grinding stone or washing hands, and re-using unwashed cooking vessels. Around 90% and 94% of opportunities (possible episodes) for handwashing with soap were missed before and during cooking, respectively (Figure 2). During cooking, around 50% of mothers also missed opportunities to cover food to protect it from kitchen dust. Table 3 shows mean TC and *E. coli* counts in 30 food samples collected immediately after cooking. High levels of TC (> 100 cfu/g) were counted in only one sample at this stage. All samples were collected within 5–15 minutes of cooking, and 77% of samples had a recorded temperature of > 60°C (mean temperature: 66°C; SD: 9).

**Figure 3.**
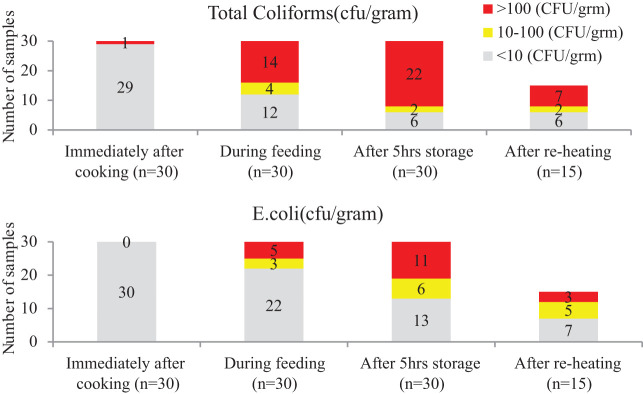
Counts of microbes in commonly used child food in different stages. This figure appears in color at www.ajtmh.org.

**Table 3:** Microbes in commonly used child food at different stages and in water and milk samples

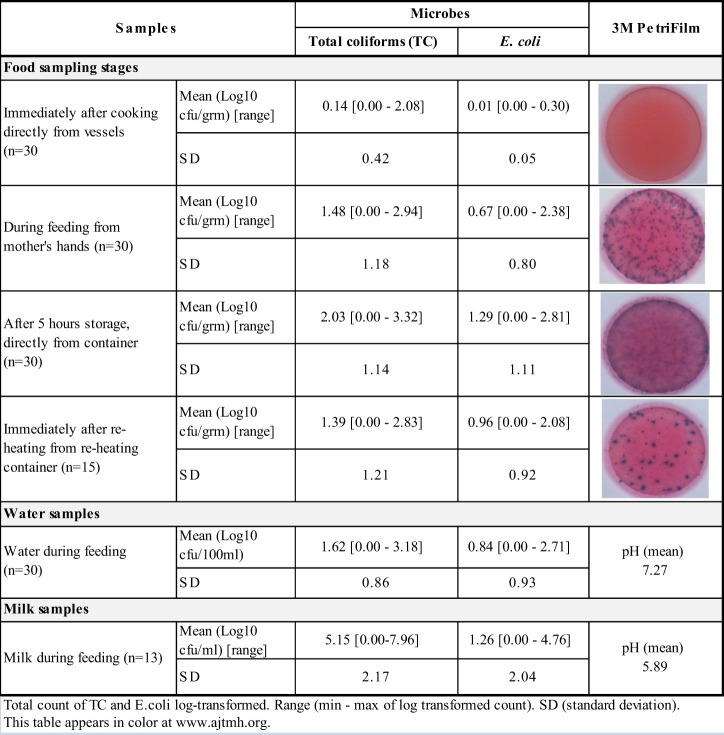

**Table 4:** Motives for practicing safe hygiene behaviors

Food hygiene practice	Prevalence (%)*	Motives for practicing/not practicing	Motive
Thorough cooking of food	77	Thoroughly cooked food test better. Following tradition to cook food only twice a day. Child wanting to eat the same family food so no separate cooking. Hot foods don’t cause stomach pain to child.	Hunger, affiliation Nurture
Cleaning of serving utensils using ash/soap	16	Cleaning utensils using ash makes them shiny and clean. Using soap removes stains and oil. Households with clean pots has higher social status. Ash is convenient, readily available, don’t have to buy it. Putting dishes on floor is convenient.	Create, disgust, status, comfort/convenience
Handwashing with soap before feeding and eating	7	Washing hands removes visible dust/dirt/bad odor and produces good smell. Child looks smart if hands are clean, and it protects their future. Handwashing with plain water outside is easy as water is readily available, but there is no handwashing station with soap in kitchen. Hands are constantly dirty from agricultural work. Soap cannot be used to wash hands in kitchen container as the soapy waste water cannot be fed to animals. Household with nice handwashing facility has higher social status.	Disgust, nurture status, comfort/convenience Hoard
Proper storage of cooked food	43	Traditional norm to use same cooking vessel or bowl/plate to store food. Covering with a lid protects food from flies/dust and protects from animals (goats, hens, cats). Flies in used spoon makes food dirty.	Affiliation, disgust
Thorough reheating of food fed to child	19	Reheated food keeps child’s abdomen warm and protects from abdominal pain. Reheated food is tastier. As food is warm in summer there is no need to reheat it. Reheating may burn it, spoil the taste, and reduce the nutritional value to the child. Relighting fire is difficult and makes smoke.	Affiliation, nurture, hunger, comfort/convenience
Boiling milk and water before feeding	0 (mid-day feeding)	Child may not like taste of boiled water. Water and milk are warm in summer so doesn’t need boiling. Reboiling milk can spoil it. Raw milk thought to be more nutritious. Boiling is easy as no extra fire needed: use leftover fire to boil water. Don’t need to be physically present to boil water like milk. Boiled water when anybody become ill. Boiled milk is tastier. Almost all believed milk is “nectar” (Amrit) and special for god to render and people to drink. Households with drinking water tap, many cows/buffalos consider as rich and has higher social status.	Hunger, nurture, affiliation, status

* Various tools were used to triangulate and best estimate the practices.

#### Cleanliness of cooking and serving utensils.

Foods were served in plates or bowls shortly after cooking, Video analysis shows around 84% of the opportunities to clean serving utensils just before offering food were missed by mothers, which was confirmed by direct observation. Nevertheless, half (47%) considered such practice as good. When dishes were cleaned, ash was used because it is readily available in all households and the use of soap was considered a luxury. Because the majority of households did not have a rack, washed utensils were stored (placed) on the mud floor. Flies, animal feces, and dust or dirt were therefore likely to contaminate washed utensils.

#### Feeding and handwashing with soap.

Observation showed that almost all mothers fed the child using their hands (81%) and that children ate with their hands. Children were often observed carrying food around while eating. Although 18% of mothers said that they believed that hands should be clean to maintain good food hygiene practices, only 7% reported the use of soap to wash their hands before cooking and before feeding their child. Soap and water were available in 94% and 100% of households, respectively. According to the video analysis, 88% of mother and 91% of child handwashing opportunities before feeding were missed by mothers. Use of water to wash hands and varying practices was commonly reported; a participating mother mentioned: “I always wash my child’s hands with water before feeding but if their grandmother is feeding, she never does this.” The second round of samples was taken during child feeding, and samples of food were collected from mothers’ and children’s hands. The mean food temperature during feeding was 37°C (SD: 6). Mean TC and *E. coli* counts were higher (Table 3, Figure 3). Of the 30 samples tested, high levels of TC (> 100 cfu/g) were counted in 14 samples (47%), and high levels of *E. coli* were counted in 5 (15%) samples.

#### Storage of cooked leftover food.

All households reported storing leftover food for subsequent feeding, and such practice was observed in 73% of households. Although 63% of households covered food for storage, the majority placed a dirty ladle over the stored food, which attracted flies, dust, dirt, and animals. Less than half (43%) covered food with a tight-fitting lid. Protecting food from flies and dust was considered good hygiene practice by only 13% and 4% of mothers, respectively, and cooked food stored up to 12 hours was considered to be fresh. It was served to children multiple times during the day. The leftover foods were either stored in the cooking vessel or in a small bowl or plate on the floor near the kitchen. After 5 hours of storage, food samples had mean temperature of 32°C (SD: 3). Mean TC and *E. coli* counts reached 2.03 log_10_ cfu/g and 1.29 log_10_ cfu/g, respectively (Table 3, Figure 3). High levels of TC (> 100 cfu/g) were counted in 22 (73%) samples, and high levels of *E. coli* were counted in 11 (37%) samples.

#### Reheating stored/leftover food before feeding.

Observation revealed that only half of households reheated stored food and that only 19% of food reheated reached an adequate temperature (> 60°C). Of those who reheated food, some used a frying pan and some used a steel serving bowl. None listed thorough reheating as a good food hygiene practice, and a statement made by one mother represents a common practice: “I do not reheat food because it will take time and energy, and it will burn the food.” The fourth batch of samples was taken from foods that had been reheated. Within 0–5 minutes of doing so, however, only 19% of samples had a temperature of > 60°C (mean: 51°C; SD: 13). The mean counts of TC and *E. coli* in were 1.39 log_10_ cfu/g and 0.96 log_10_ cfu/g, respectively. Of the 15 samples tested, high levels of TC and *E. coli* (> 100 cfu/g) were counted in 7 (23%) and 3 (10%) of the samples.

#### Water and milk feeding.

Young children were always offered either water or home-produced cow/buffalo milk together with, or immediately after, food. Water from communal or household taps (delivered from unprotected sources) was commonly collected in thin copper or brass vessels that were observed uncovered (47%) or covered with a bowl (53%). None of the households boiled water before serving it to the children, and none of them reboiled milk when serving it at different times during the day. As revealed from video footage, all water treatment and 95% of milk boiling opportunities were missed by mothers. Though milk was usually boiled on receipt/after milking, a few households gave their children never-boiled milk to drink. Water, milk, and Jad samples were collected during mid-day. The mean TC and *E. coli* counts in 30 water samples collected during feeding were 1.62 log_10_ cfu/100 mL and 0.92 log_10_ cfu/100 mL, respectively. Milk samples were heavily contaminated with TC and *E. coli*, with mean counts of 5.15 log_10_ cfu/mL and 1.26 log_10_ cfu/mL, respectively, with a mean pH of 5.9. Twelve Jad samples were tested, and no TCs or *E. coli* were isolated (mean pH of 2.9).

### Hazard analysis and determination of control points.

**Figure 4.**
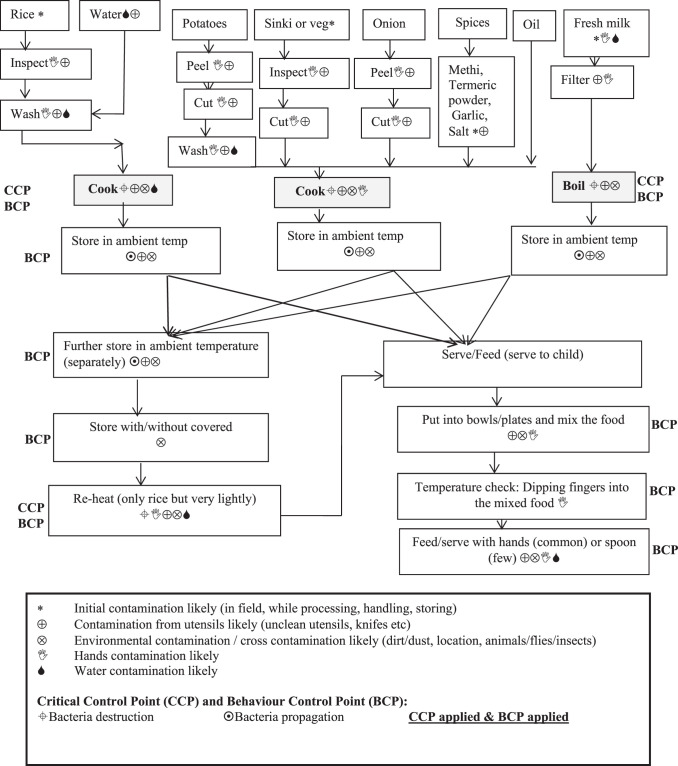
Food flow diagram of commonly used child food: likely hazards, critical and behavioral control points.

A food flow diagram from collection of raw materials to cooking to storage to feeding was drawn, and potential sources of hazards and possible re/contamination were documented for each key step (Figure 4; Table 5). Critical control points were identified based on their role in bacteria destruction, survival, and/or propagation using microbial evidence (Table 3). Behavioral control points were identified depending on whether behavioral actions could be applied as control measures (Figure 4; Table 6).

**Table 5:** Hazard analysis: rice, vegetable, and milk processing and potential sources of contamination

Steps	Possible source of contamination
Rice from the home/market	Field, processing and handling, storage container
Inspect rice	Hands, utensil, environmental exposure (dirt/dust)
Wash rice and discard water	Container to collect rice, water, hands, utensil, dirt and dust
Cook rice	Utensil, time, temperature, water, duration, location, fuel used
Store rice	Cover, location, animal exposure, ambient temperature, utensil, flies
Vegetables from kitchen garden/market	Field, utensil, hands, environmental exposure
Wash and cut vegetables	Water, bowl/plates, knife, chopping board, flies
Put oil in the pot (oil from market)	Utensil, hands, environmental contamination (dirt/dust), smoke/ash
Grind coriander seeds, turmeric powder, chilli, garlic, ginger, etc. in the stone	Contaminated spices, hand and utensil contamination to peel and cut garlic/ginger, rock and mortar, water, flies
Cook vegetables	Spices (Masala), water, utensil, temperature, duration, location, environmental exposure, cover, flies
Add water and salt	Water, water vessel, salt, temperature, salt container
Stir and take out from stove	Spoon, cover, animal exposure, environmental exposure
Store curry	Cover, location, animal exposure, ambient temperature, utensil, flies
Milking cow/buffalo	Milking container, hands, water, cow/buffalo, location, flies/insects
Milk processing	Filter (rare practice) using sieve or cloth, boil temperature, utensil
Milk storage	Inadequate cover, environmental exposure (flies, dirt), storage utensil, animal exposure,
Wash hands before feeding	Hands, water, techniques
Serve food with curry or milk	Utensils/spoon, plates/bowls, water, hands, glass
Feeding to child	Mother’s hands, child’s hands, spoon, bowls/plates, environmental, water, milk, jad, flies
Store with cover or without cover	Duration, location, inadequate cover, flies/insects, animal exposure, dirt/dust, ambient temperature
Briefly reheats/no re-heat before feeding	Duration, utensils, temperature, hands
Feeding reheated or cold food	Hands, spoon, bowls/plates, environmental, water, milk, jad, flies

**Table 6:** Five key prioritized behaviors and reasons for promotion

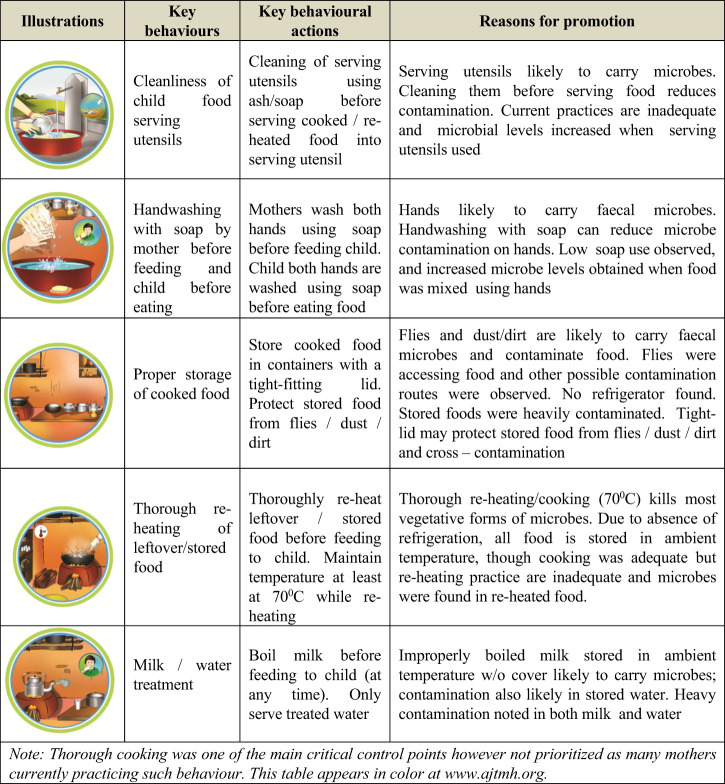

This analysis identified initial cooking, later reheating of food, and boiling of water and milk as critical control points through which bacterial destruction could be expected. Further critical control points concerned storage, use of serving utensils, and feeding/eating using hands or spoon. Corrective behavioral control measures include proper storage of cooked food with a tight lid, cleaning of serving utensils (plates, bowls, and spoons) using ash or soap, and handwashing with soap before feeding and eating.

### Environmental determinants of food hygiene behavior.

#### Social environment.

Social factors that influenced food hygiene behavior included ethnicity, contact with health workers, and contact with the outside world. In the Hindu Brahmin/Chhetri group, cow dung was smeared on kitchen floors after eating every day with a belief that cow dung will purify the places after eating. Among the Tamang it was the norm to only wash cooking and serving utensils once a day in the late morning. A wooden spoon was used to mix flour while cooking dhindo, but it was important to them that it never be washed; instead, remnants were peeled or scraped off before use. In both Brahmin and Tamang, it was the norm to only wash the milk storage container twice a month. The majority of Tamang families also fed the alcoholic drink Jad to children under 5 years of age as a substitute for water and milk. A mother from this ethnic group explained: “I feed Jad to the child so that they don’t cry and disturb my work; at the same time, Jad kills the worms in the child’s stomach.” Apart from family and local community, the sources of new information were reported as teachers and female community health volunteers. Most mothers attended monthly credit group meetings and listened to local radio during leisure time, but few had contact with television.

#### Physical environment.

The physical components of the settings presented a challenging environment for food hygiene. Most households had piped water connections, but the water was untreated, around 57% practiced open defection, and none had refrigerators. All houses were made of mud and stone, with kitchen surfaces made of mud, and most households cooked on the floor using firewood. Most of the households did not have a separate kitchen; hence, one room on the ground floor served for cooking, eating, running of the household, sleeping, and sometimes for keeping animals such as chickens, goats, and buffaloes. This room was often dark and smoky. Only 7% of households had a cupboard in which to store washed kitchen utensils. Figure 1 shows the overall cleanliness status of the study settings.

#### Biological environment.

Animal feces were observed on the ground in 79% of compounds, and the majority (65%) of households disposed of child feces in adjacent fields or open spaces. Flies were present throughout the village, particularly in kitchens, making contamination likely. Only 34% of compounds, 45% of toilets, 57% of the kitchens, and 67% of the stored utensils was classed as visibly “clean” (Figure 1). Almost all households collected waste kitchen water to feed animals that attracted flies. They did not wash hands with soap in this container in kitchen because they believe that animals should not be given soapy water. Only 28% of children’s and 40% of mother’s hands were classed as visibly “clean.”

#### Beliefs about diarrhea.

The reported prevalence of diarrhea in children in the past week was 24%. When asked what they thought caused diarrhea, one-third of mothers mentioned flies and dust, and a quarter mentioned contaminated food. Other causes included were not washing hands with soap before feeding child and the “evil eye.” In FGDs, mothers reported that they first seek the services of traditional healers when their child had diarrhea, and only if the situation becomes worse do they seek support from female community health volunteers or take the child to a nearby health institution.

### Psychological determinants (key motives) of food hygiene behavior.

The psychological determinants including immediate motives and the motivational drivers for key behaviors, such as cooking food thoroughly, cleaning serving utensils using ash or soap, handwashing in the kitchen before feeding/eating, covering food, reheating stored food before feeding, and treating water or milk before feeding are reported in Table 4. Mothers were predetermined to perform routine food hygiene behaviors in their respective environmental settings as they are traditionally practicing some common actions in their daily life. The understanding of behavioral determinants and identification of barriers was a basis to explore what would motivate mothers to address some of those barriers through the motivational exercise using an imaginary story. Mothers reported almost similar motives to practice a specific behavior but different sets of motives for different behaviors. The key emotional drivers were linked with specific motives linked with specific behaviors. After analysis, we found that “nurture, disgust, affiliation, and status” were the common motivational drivers to practice food hygiene behaviors. The key motives and their drivers are presented in Table 4.

## DISCUSSION

This study showed that the prevalent food hygiene behaviors of mothers were inadequate as assessed using multiple methods. Optimal behaviors that would mitigate fecal oral transmission of microbes in commonly used child food, such as cleanliness of serving utensils, handwashing with soap before feeding/eating, proper storage, and reheating and boiling milk/water before offering were uncommon among mothers (Figure 2; Table 4). Young children in rural Nepal were exposed to microbes in food, water, and milk. Although various factors and multitasking behaviors of mothers were thought to be responsible for re-/contamination of food, water, and milk, the HACCP approach was useful to identify six critical and behavioral control points. The identification of critical control points is particularly important and can facilitate appropriate targeting of resources and prevention efforts.^32^ The immediate motives and emotional drivers for practicing behaviors were identified for each behavior.

Our findings showed that the physical settings, especially the kitchen, present a challenging environment for mothers to properly practice adequate food hygiene behaviors. Pertinent aspects of the physical environment included the lack of proper kitchen settings, the lack of infrastructure, and difficulty in meticulously cleaning the kitchen surfaces compounded by an unclean household environment. This is compounded by the biological environment (heavy presence of flies, animals accessing in kitchen, presence of animal feces in compound, etc.) and the social environment (different ethnic groups followed different practices) and traditions (feeding family food to children, etc.). A review article demonstrates that weaning foods prepared under unhygienic conditions are frequently heavily contaminated with pathogens and thus are a major factor in the cause of diarrheal diseases and associated malnutrition.^17^ In Vietnam, the risk of diarrhea differed by cooking places.^33^ Our study suggests that food hygiene behaviors are underpinned by physical, social, and biological settings. Initiatives to improve food hygiene behaviors in such environments should consider changes in physical, social, and biological settings, most importantly the kitchen setting, though it would be challenging.

Thorough cooking was identified as a critical control point when appropriate temperature is maintained. Our findings show that food may have become contaminated/recontaminated on multiple occasions during cooking due to multitasking by mothers during cooking. Despite these risks, thorough cooking practices eliminated microbial contamination (TC and *E. coli*) risks even though mothers were unaware of the benefits of thorough cooking. Thorough cooking is one of the most important critical control points if a high level of temperature (> 60°C) is maintained, which kills vegetative forms of pathogenic bacteria.^34^ Previous studies using HACCP also found that traditional cooking was very effective in eliminating fecal contamination.^35^ Reinforcement and encouragement using immediate motives would be useful for sustaining thorough cooking practices.

Handling food increases the risk of contamination. Our hazard mapping showed that the foods were subject to contamination during serving and feeding/eating, yet many mothers missed opportunities to properly wash their hand or the child’s hands and serving utensils before serving food. Food samples taken immediately after cooking from plates or bowls after hand contact showed increased levels of contamination. This shows that promoting thorough cooking alone is insufficient to prevent microbial contamination at the point of feeding because additional contamination is likely from hands and serving utensils during this time if handwashing is not practiced. A main reported and observed barrier for not washing hands in the kitchen was that all households collect kitchen wastewater in a container that is then fed to animals. Families feared that if they used soap to wash their hands, the animals would not drink the soapy water. Hence, mitigating this barrier through the placement of specific handwashing facilities in the kitchen setting may increase the frequency of handwashing during cooking and before feeding the child. Mothers gave other various reasons, which are presented as “verbatim” in Table 7. Although handwashing with soap can prevent infection and save many lives, it is still rare in many countries,^26^ and handwashing before feeding children and before handling food was practiced less than handwashing at other times.^36^ We suggest that promotion of food hygiene should include cleanliness of serving utensils and handwashing with soap before feeding and eating. Previous studies suggest that mothers quickly adopt handwashing with soap as compared with spoon-feeding practices.^37^ The immediate motives for practicing handwashing and cleanliness of utensils might be useful to reinforce such practices.

**Table 7:** Verbatim in relation to “handwashing at different times” and “reasons for not reheating or heating the stored food” Mothers gave many reasons during formative research why they should wash hands, why not and difficulties, and why not reheat food and few reasons why they should. The following table gives some of the verbatim quotes from the mothers

Verbatim in relation to handwashing at different times	
Verbatim related to “why to wash hands”	Verbatim related to “why not and difficulties”
“I wash my hands with water if I see any dirt/dust in my hands.” “I wash my hands with soap after cleaning utensils because my hands would be black if I don’t use soap after cleaning them.” “If we wash hands our children will also learn that behavior.” “I wash hands because it removes the dirt and germs.”’ “Unless we put water in the hands before eating/feeding we are not satisfied. This is our tradition.” “We have a child who had difficulties to walk, speak and eat. I have to wash his hands with water before he eats food. We don’t have money to buy the soap.” “Washing hands with soap before cooking and feeding child can ensure child's food safety in the kitchen.” “'I always wash child's hands with water before feeding to baby but if grandmother is feeding, she never does this.”	“In kitchen we don’t use soap because we feed waste water to animals, if we use soap they will not drink. I told everyone to wash hands out if needed?” “Sometimes do not get the time to wash hands, sometimes there is no soap in the house, sometimes there is no money to buy soap, and sometimes forget to wash the hands.” “I always use water to wash hands, but findings soap always is quite difficult.” “I wash my hand before feeding the child, so why to wash child’s hand before feeding.” “We have soap, but not everyone in the house uses it.” “When I washed my hands and feet with soap and water it caused allergy, so I wash my hands and feet with ash and water.” “What’s the harm of washing hands with mud or cow dung if there is no soap and ash? If available, I prefer ash, but young daughters wants soap.”
Reasons for not re-heating or heating the stored food	
Why to reheat food?	Reasons for not reheating the food
“Hot food is good for the health, and it doesn’t cause stomach pain.” “In winter, it is nice if the food is warm.” “Reheated food is always tasty, and child will eat more.”	“No one comes in time to eat food, and child wants food frequently, so how many times to reheat the cooked food?” “I don’t reheat because it will take time and energy and it will burn.” “If I reheat food, all nutritious value will destroy and child will have diarrhea after eating that food, it is therefore, I won’t re-heat the food.” “If we reheat cooked food long, it will be sticky, and all nutrients value will go away.” “For long reheated food, we considered as leftover food; hence, I always briefly reheat if I had to do so in cold season. That way it will also be quicker to feed child straight away.” “Who bothers to reheats the food? Child eat the cold food by themselves at any times without our any support.” “In winter we reheat the food before eating, but in summer if the food is reheated it becomes sour.” “If I cook food in the morning, it will be sufficient up to evening for child. I don’t reheat the food in summer because child can’t eat hot food.” “I just briefly reheat the food because no one can eat the hot food.” “If child starts crying, I can immediately provide leftover food.”

Leftover cooked food was kept at ambient temperatures after the first feeding because none of the households had a refrigerator, making the multiplication of organisms likely. Food samples tested after 5 hours of storage had a higher level of contamination compared with any other time. Lukewarm temperature, the environment (flies/dust/dirt/storage location), inadequate utensil storage, and storage without a tight-fitting lid all make these high levels of contamination possible. Previous studies also reported increased bacteria counts from 10^4^ to > 10^8^ after 24 hours at 37°C,^38^^–^^42^ which is a threefold increased within 1 hour of storage,^40^ and significant multiplication of fecal coliforms when there was a delay of more than 4 hours between preparation and consumption of food.^43^ Avoiding storage of cooked food was difficult in the study setting; hence, storage of leftover food with a tight-fitting lid to avoid additional contamination from flies, dust and dirt, and animals is the only possible measure that can be applied at this stage in the study settings. The identified immediate motives would be a useful key message to encourage this habit.

Offering stored food without reheating or briefly reheating was common in the study setting, and various barriers to reheat food were reported. Bacteria were not killed in sufficient quantities in briefly reheated food, suggesting that reheating practices were inadequate and temperature had a major role in killing bacteria. The practice of touching food with hands, adding water to make food wet, and not maintaining proper temperature also add more potential contamination. The cauldron was identified as an appropriate object for thoroughly reheating food, yet many households did not use it for this purpose. Thorough reheating of stored food just before offering to the child was identified as a control point, and immediate motives to practice thorough reheating would be used as key messages to encourage this habit.

None of households treated water before serving to children, and the majority of water samples were contaminated with TCs and failed to meet WHO and Nepal national water quality standards.^44^ Contamination was likely caused by contamination at the source (unprotected spring), storage practices (uncovered containers), and contaminated serving utensils (glasses or bowls). The kettle was identified as an appropriate object with which to boil water but was mostly used for tea preparation. Similarly, milk was also heavily contaminated, due to unhygienic milking (location, washing gutter using contaminated water, etc.), inadequate boiling, inadequate storage (storing in ambient temperatures without covering), inadequate handling, and not reboiling before serving. The most feasible options identified to make water and milk safe were boiling in that setting at the household level. Boiling water was identified as one of the preferred household water treatment options, and its effectiveness was previously tested.^45^ The motives identified to boil water and milk just before feeding child could encourage such practice. Jad had nondetectable levels of TC, and *E. coli* and all Jad samples were more acidic, which might have affected the level of microbiological contamination.

While identifying the control points, it was recognized that each of the points can offer critical control measures (killing bacteria) and behavioral control measures (reducing contamination). The food flow diagram and mapping show that cooking/boiling, storing, and reheating food offers the best possible critical control points. In addition to critical control points, this study identified behavioral control points in between cooking and feeding practices; failure to address these control points in fact increases the level of contamination in food either through food serving utensils (plates, bowls) or through hands.

Behavioral assessment, identification of determinants, assessment of microbiological contamination in food, and finally identification of critical and behavioral control points offered sufficient information for prioritizing multiple food hygiene behaviors to address all transmission pathways in the next phase, focusing on critical control and behavioral control points. We suggested the following six key behaviors should be prioritized as control measures for behavior action: 1) thorough cooking of child food; 2) cleaning serving utensils using ash or soap just before serving cooked or reheated food; 3) handwashing with soap before feeding and before eating; 4) proper storage of leftover cooked food with a tight-fitting lid to protect from flies, dust, or dirt; 5) thorough reheating of any stored/leftover food just before feeding child; and 6) treatment of water and milk before serving to a child.

To design and implement a food hygiene promotion intervention, we recommend prioritizing five key behaviors because the majority of mothers already practice thorough cooking in this setting, a practice socially and culturally rooted in the rural settings. Details and rationale regarding the five prioritized behaviors are presented in Table 6.

Our formative research had several limitations. Due to the qualitative nature of the study, it was not possible to perform statistical analysis and establish causal associations with the contributing factors. We have best estimated the behavioral outcomes using multiple tools rather than structured observation for all behaviors following one identical method. To best feed the information for the design of the intervention (for next phase), we have explored in-depth understanding and further probed the causes of practicing adverse behaviors that might introduce a certain level of bias, but the use of different tools triangulated the outcomes in various ways. Our evidence may not be generalizable to the urban context of Nepal. The samples were taken during the pre-monsoon season; therefore, the microbes presented in this study may be lower than would be typical during the wet seasons in such a contaminated environment. We have only included TCs and *E. coli* as indicators of presence of fecal matter in food, water, and milk, but it would have been ideal to analyze the presence of fecal pathogens and human-specific bacteriodale species by using sensitive molecular techniques. Such limitations do not negate the implications of this study.

Evidence is mounting that current efforts are insufficient to prevent diarrheal diseases in low-income settings; this may be because most programs exclude food hygiene interventions and therefore fail to address critical transmission pathways. Our findings suggest that current WASH interventions will not effectively eliminate the fecal–oral transmission of microbes unless control measures are applied in these points. The HACCP^23^ approach, including anthropological and consumer research techniques, was useful to identify six critical and behavioral control points and to prioritized five key adversely practiced behaviors that are suggested for prioritization as a control measure and for the design of an intervention. Our next step will be to design a simple and scalable food hygiene intervention targeting key prioritized behaviors for cost-effective implementation in normal community settings using behavior change principles/approaches. The study also concluded that, when designing a food hygiene intervention package, consideration should be given to slight changes in the physical, biological, and social environment, particularly the kitchen. The immediate motives behind each practice should be taken into consideration while framing key messages. The specific tools should be designed around common motivational themes as drivers of behavior change such as nurture, disgust, affiliation and social status/respect and the design of a food hygiene intervention trial should use them.^46^ This study also tested the detailed methodology to conduct formative research on food hygiene in rural settings that can be applied in other low-income settings.
